# FKBP8 Enhances Protein Stability of the CLC-1 Chloride Channel at the Plasma Membrane

**DOI:** 10.3390/ijms19123783

**Published:** 2018-11-28

**Authors:** Yi-Jheng Peng, Yi-Ching Lee, Ssu-Ju Fu, Yun-Chia Chien, Yi-Fan Liao, Tsung-Yu Chen, Chung-Jiuan Jeng, Chih-Yung Tang

**Affiliations:** 1 Department of Physiology, College of Medicine, National Taiwan University, Taipei 10051, Taiwan; yijhengp@usc.edu (Y.-J.P.); angela2747@yahoo.com.tw (Y.-C.L.); d01441001@ntu.edu.tw (S.-J.F.); wingtalk1006@hotmail.com (Y.-C.C.); 2 Institute of Anatomy and Cell Biology, School of Medicine, National Yang-Ming University, Taipei 10051, Taiwan; cassis0211@hotmail.com; 3 Neuroscience Center, University of California, Davis, CA 95616, USA; tycchen@ucdavis.edu; 4 Brain Research Center, National Yang-Ming University, Taipei 12212, Taiwan; 5 Graduate Institute of Brain and Mind Sciences, College of Medicine, National Taiwan University, Taipei 10051, Taiwan

**Keywords:** ion channels, molecular chaperones, membrane proteins, protein stability, trafficking, skeletal muscle

## Abstract

Mutations in the skeletal muscle-specific CLC-1 chloride channel are associated with the human hereditary disease myotonia congenita. The molecular pathophysiology underlying some of the disease-causing mutations can be ascribed to defective human CLC-1 protein biosynthesis. CLC-1 protein folding is assisted by several molecular chaperones and co-chaperones, including FK506-binding protein 8 (FKBP8). FKBP8 is generally considered an endoplasmic reticulum- and mitochondrion-resident membrane protein, but is not thought to contribute to protein quality control at the cell surface. Herein, we aim to test the hypothesis that FKBP8 may regulate CLC-1 protein at the plasma membrane. Surface biotinylation and subcellular fractionation analyses reveal that a portion of FKBP8 is present at the plasma membrane, and that co-expression with CLC-1 enhances surface localization of FKBP8. Immunoblotting analyses of plasma membrane proteins purified from skeletal muscle further confirm surface localization of FKBP8. Importantly, FKBP8 promotes CLC-1 protein stability at the plasma membrane. Together, our data underscore the importance of FKBP8 in the peripheral quality control of CLC-1 channel.

## 1. Introduction

In skeletal muscles, up to 80% of the resting membrane conductance is determined by the voltage-dependent CLC-1 chloride (Cl^−^) channel [1,2,3], indicating that the Cl^−^ channel is essential for setting the resting membrane potential after firing of action potentials. Mutations in the human gene (*CLCN1*) encoding CLC-1 channels have been linked to the hereditary skeletal muscle disorder myotonia congenita, which manifests delayed muscle relaxation following voluntary contraction [4,5,6,7]. Whereas many disease-associated mutations entail altered channel gating functions [8,9,10,11], some other mutations lead to disruptive biosynthesis of CLC-1 channels [12,13,14,15].

The human CLC-1 mutant A531V, a myotonia congenita-causing mutation [16,17], is associated with substantially reduced whole-cell current density that can be attributed to enhanced proteasomal and lysosomal degradations, as well as defective membrane trafficking [12,13]. Previous studies from our lab further demonstrate that, at the endoplasmic reticulum (ER), CLC-1 protein is subject to an ER-associated degradation mechanism mediated by the Cullin 4A/B E3 ubiquitin ligase complex [18]. Moreover, the ER quality control system for CLC-1 protein involves the molecular chaperones heat shock cognate protein 70 (Hsc70) and heat shock protein 90β (Hsp90β), as well as the co-chaperones Hsp70/Hsp90 organizing protein (HOP), activator of Hsp90 ATPase homolog 1 (Aha1), and FK506-binding protein 8 (FKBP8; also known as FKBP38) [19], many of which have also been shown to take part in ER quality control of other proteins such as cystic fibrosis transmembrane conductance regulator (CFTR) Cl^−^ channel and human ether-à-go-go-related gene potassium (K^+^) channel [20,21,22,23,24,25]. Importantly, our data suggest that Hsp90β and FKBP8 may play a central role in ER quality control of CLC-1 protein by dynamically coordinating with the Cullin 4A/B ubiquitin ligase complex. 

The nature of the molecules governing CLC-1 protein quality control at the plasma membrane, however, remains elusive. Among the CLC-1-interacting ER molecular chaperones and co-chaperones we identified, FKBP8 is unique in that it is a membrane-resident protein [26,27], and the co-chaperone displays the most significant effect in enhancing CLC-1 protein expression at the plasma membrane [19]. This prominent increase in surface expression implies that, at the ER, FKBP8 is distinctively effective in facilitating membrane trafficking of CLC-1 protein. Alternatively, the data can be interpreted as that FKBP8 may play an active role in the protein quality control of CLC-1 at the plasma membrane, thereby notably improving surface protein stability of the Cl^−^ channel. Nonetheless, to date there is no direct evidence supporting plasma membrane-localization of FKBP8. In this study, we aim to address this critical issue by applying various biochemical approaches to investigate whether FKBP8 and CLC-1 may be co-localized at the plasma membrane. Overall, our results are consistent with the idea that FKBP8 may directly contribute to the maintenance of CLC-1 protein quality at the cell surface.

## 2. Results

### 2.1. Surface Biotinylation of FKBP8

**Figure 1:**
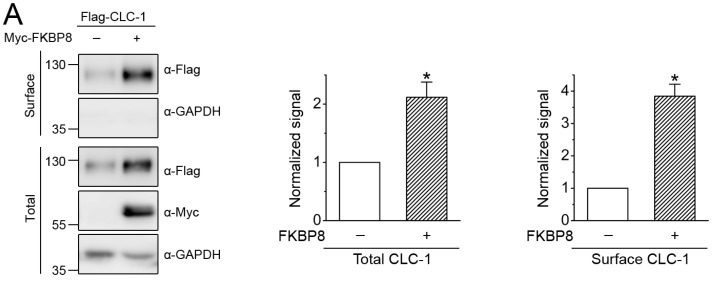

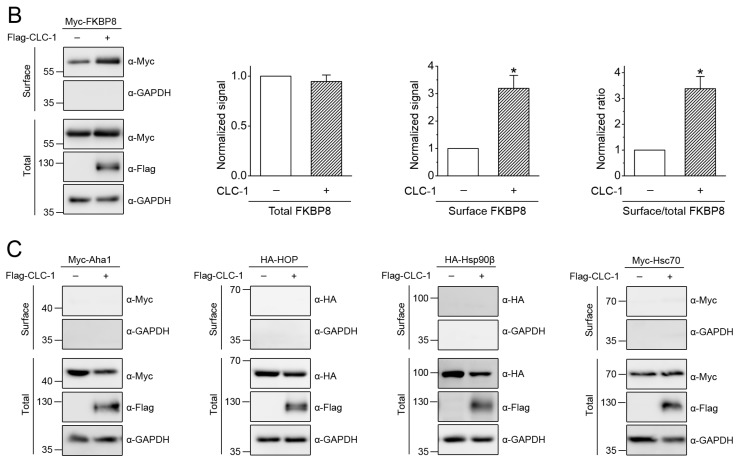
Detection of cell surface FKBP8 expression by biotinylation. (**A**) Surface biotinylation experiments on HEK293T cells expressing Flag-CLC-1 in the absence or presence of Myc-FKBP8. (*Left*) Representative immunoblots. Co-expression with the Myc vector was used as the vector control (-). Cell lysates from biotinylated intact cells were subject to either direct immunoblotting analyses (*Total*) or streptavidin pull-down prior to immunoblotting (*Surface*), using the indicated antibodies (α-Myc, α-Flag, or α-GAPDH). Total represents about 8% of the amount of the protein used for streptavidin pull-down. The molecular weight markers (in kDa) are labeled to the left. GAPDH expressions are shown as the loading control. (*Right*) Quantification of total and surface CLC-1 protein levels (*n* = 6). The protein density was normalized to that of the corresponding vector control; (**B**) Surface biotinylation analysis of Myc-FKBP8 in the absence or presence of Flag-CLC-1. (*Left*) Representative immunoblots. (*Right*) Quantification of FKBP8 protein levels and membrane trafficking (*n* = 8). The membrane trafficking efficiency of FKBP8 was expressed as surface protein density divided by the corresponding total protein density (*Surface/total*); (**C**) Representative immunoblots showing surface biotinylation analysis of Myc-Aha1, HA-HOP, HA-Hsp90β, or Myc-Hsc70, in the absence or presence of Flag-CLC-1. Asterisks denote significant difference from the control (*, *t*-test: *p* < 0.05).

By performing co-immunoprecipitation and glutathione S-transferase (GST) pull-down assays, we previously showed that FKBP8 is a binding partner of human CLC-1 channel [19]. Furthermore, in line with our prior surface biotinylation studies, Figure 1A shows that co-expression with Myc-tagged FKBP8 (Myc-FKBP8) promoted both total and surface expressions of Flag-tagged CLC-1 (Flag-CLC-1) in HEK293T cells. To address the possibility that FKBP8 may be present at the plasma membrane, we began by investigating whether FKBP8 can be identified by surface biotinylation assay. Surprisingly, a notable fraction of Myc-FKBP8 was indeed detected by surface biotinylation (Figure 1B, *left panel*). Moreover, co-expression with Flag-CLC-1 resulted in about 3-fold increase in the surface signal, but not the total protein level, of Myc-FKBP8 (Figure 1B), suggesting that the presence of CLC-1 may enhance cell surface level of FKBP8. 

In addition to FKBP8, we have demonstrated that the ER quality control system for CLC-1 protein also involves the co-chaperones and molecular chaperones Aha1, HOP, Hsp90β, and Hsc70 [19]. Interestingly, Hsp90 and Hsp70 were found to be present at the cell surface of some tumor cells and developing neurons [28,29,30,31]. We therefore repeated the same biotinylation experiments with Aha1, HOP, Hsp90β, and Hsc70 to explore their potential cell surface localization in our heterologous expression system. Figure 1C clearly indicates that, regardless of whether CLC-1 was present, no discernible surface biotinylation signal was found for the other CLC-1-interacting co-chaperones and chaperones. This negative result, however, does not exclude the possibility that some of these molecular chaperones and co-chaperones may be associated with CLC-1 at the plasma membrane.

### 2.2. Localization of FKBP8 at the Plasma Membrane and the Golgi Complex by Subcellular Fractionation

In the following sets of experiments, we addressed the subcellular localization of FKBP8 and CLC-1 by applying a variety of different fractionation analyses. We began by employing sucrose density gradient centrifugation whereby lipid-soluble organelles were separated into 8 distinct fractions on the basis their relative densities, with the density gradient increasing from fraction 1 toward fraction 8. Endogenous glyceraldehyde 3-phosphate dehydrogenase (GAPDH), calnexin, and cadherin in HEK293T cells were used as the internal control for differentiating cytosolic, ER membrane, and plasma membrane proteins, respectively. Calnexin is principally localized at two sub-domains of the ER, the rough ER and the mitochondria-associated membrane [32,33,34], both of which are associated with a higher density relative to the ER-Golgi intermediate compartment as well as the Golgi complex. Given the fact that FKBP8 is typically considered as an internal membrane protein resident at the ER and the mitochondria [26,35,36,37], it is likely that FKBP8 and calnexin share a similar localization pattern within the ER.

**Figure 2:**
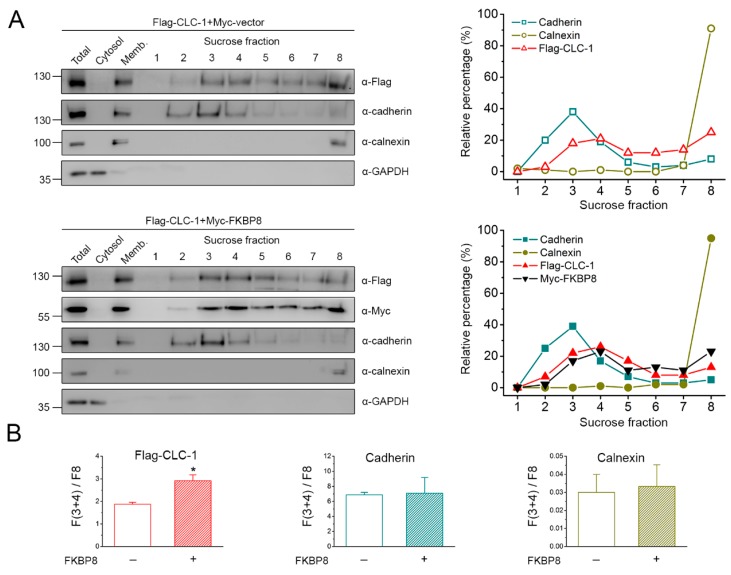

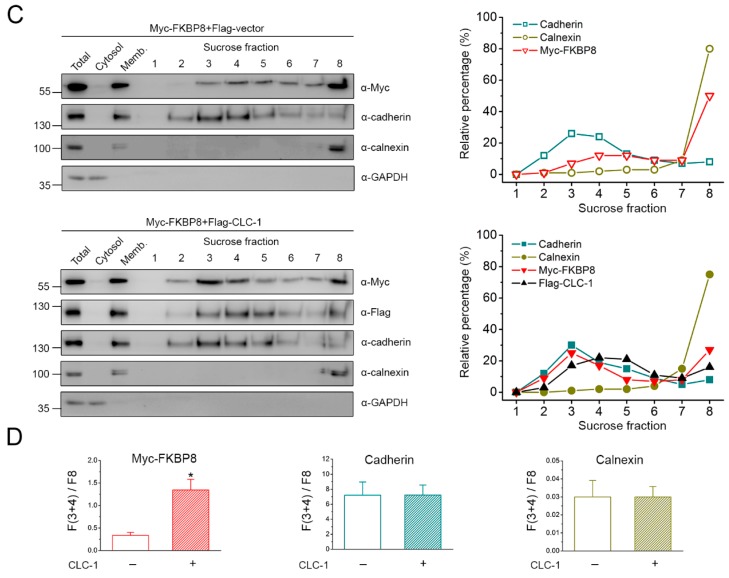
Sucrose gradient fractionation analyses of FKBP8 localization. Cell homogenates from HEK293T cells transfected with the indicated cDNA constructs were analyzed by sucrose density gradient centrifugation. Total homogenates (*Total*) were ultracentrifuged and separated into the supernatant (*Cytosol*) and the membrane pellet (*Memb*) fractions. The membrane pellet fraction was further sedimented through a discontinuous sucrose gradient and subsequently divided into 8 fractions, with the density gradient increasing from fraction 1 toward fraction 8. Specific markers for distinct subcellular compartments were chosen by studying 3 endogenous proteins in HEK293T cells: cadherin (plasma membrane protein), calnexin (ER-resident membrane-associated protein), and GAPDH (cytosolic protein). (**A**) (*Left panels*) Representative immunoblots showing the subcellular fractionation pattern of Flag-CLC-1 in the absence or presence of Myc-FKBP8 co-expression. (*Right panels*) Densitometric quantification of the relative distribution (with respect to the total signal) in different fractions for each membrane-associated protein analyzed on the corresponding gel to the left; (**B**) Statistical analyses of the ratio of protein signal intensities at fractions 3 and 4 to those at fraction 8 [F(3+4)/F8] for the indicated membrane-associated proteins in the absence or presence of Myc-FKBP8 (*n* = 3); (**C**) Representative sucrose gradient fractionation pattern of Myc-FKBP8 in the absence or presence of Flag-CLC-1 co-expression; (**D**) Statistical analyses of the F(3+4)/F8 ratio for the indicated membrane-associated proteins in the absence or presence of Flag-CLC-1 (*n* = 4). The asterisk denotes a significant difference from the control (*, *t*-test: *p* < 0.05).

Figure 2A shows that GAPDH was virtually undetectable at any of the 8 fractions, and that calnexin was preferentially localized at the densest fraction 8. By contrast, cadherin was detected mainly at fractions 2–4. Similar to the distribution pattern of cadherin, over-expressed Flag-CLC-1 was identified at fractions 3–5; nonetheless, Flag-CLC-1 also displayed a significant localization at fractions 7–8. Therefore, these observations indicate that, in our sucrose gradient separation system, fraction 8 most likely corresponds to ER-resident membrane proteins, whereas fractions 3–4 comprise mainly plasma membrane-resident proteins. To quantitatively evaluate the relative propensity for plasma membrane localization of these proteins, we then calculated the ratio of protein signal intensities at fractions 3 and 4 to those at fraction 8 [F(3+4)/F8]. As outlined in Figure 2B, the mean F(3+4)/F8 ratios for calnexin, Flag-CLC-1, and cadherin were about 0.03, 1.87, and 6.87, respectively. The substantial difference between the F(3+4)/F8 ratios of Flag-CLC-1 and cadherin is consistent with the common notion that, compared to their endogenous counterparts, over-expressed plasma membrane proteins are associated with a higher degree of ER retention. Moreover, upon co-expression with Myc-FKBP8, Flag-CLC-1 exhibited concurrent reduced ER localization and enhanced plasma membrane localization (Figure 2A), leading to about a 56% increase in the mean F(3+4)/F8 ratio (~2.92) (Figure 2B). By contrast, no discernible change was found for the F(3+4)/F8 ratios of cadherin and calnexin (Figure 2B).

Next, we characterized the subcellular localization of Myc-FKBP8 with sucrose density gradient centrifugation. As depicted in Figure 2C, in the absence of CLC-1, the strongest Myc-FKBP8 signal was detected at fraction 8. Unlike the fractionation pattern of the ER-resident protein calnexin, however, weak Myc-FKBP8 signals were also found at fractions 3–4 (Figure 2C). Indeed, the mean F(3+4)/F8 ratio of Myc-FKBP8 was about 0.34, which is substantially larger than that of calnexin (~0.03), albeit much smaller than that of cadherin (~7.21) (Figure 2D). Importantly, in the presence of Flag-CLC-1 co-expression, Myc-FKBP8 signals were remarkably enhanced at fractions 3–4 (Figure 2C; see also Figure 2B), leading to about a 4-fold increase in the mean F(3+4)/F8 value (~1.35) (Figure 2D). By contrast, no measurable difference was detected for the F(3+4)/F8 ratios of cadherin and calnexin (Figure 2D). Together, the preceding sucrose gradient centrifugation results are consistent with the idea that a small but significant fraction of over-expressed FKBP8 is localized at the plasma membrane, and that CLC-1 may induce a redistribution of ER-resident FKBP8 to the plasma membrane.

**Figure 3:**
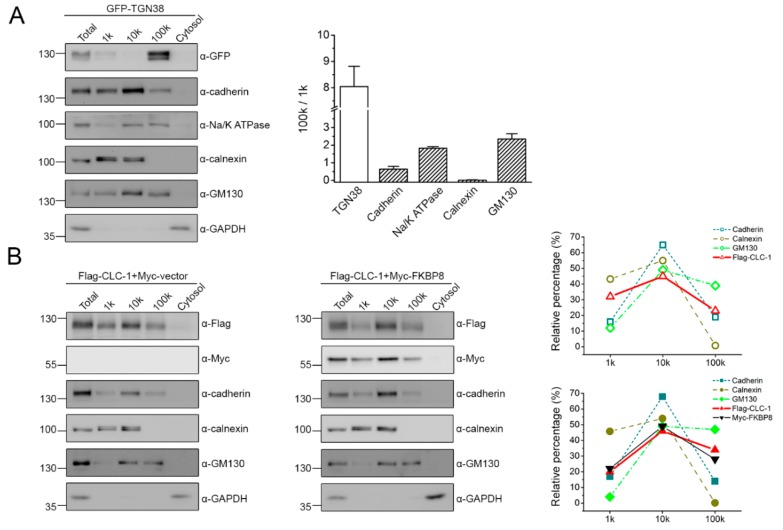

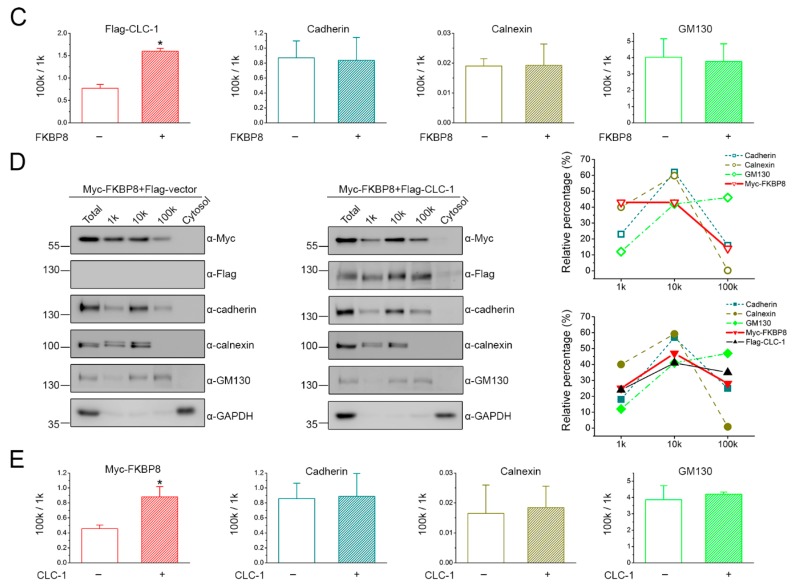
Localization of FKBP8 at the Golgi apparatus. (**A**) Differential centrifugation analysis of lysates from HEK293T cells over-expressing GFP-tagged *trans*-Golgi network 38 (GFP-TGN38). (*Left*) Representative immunoblots showing the fractionation pattern of GFP-TGN38 and the indicated endogenous proteins. Total homogenates (*Total*) were separated by sequential 1000× *g* (1 k) and 10,000× *g* (10 k), and 100,000× *g* (100 k) centrifugation. The pellets retrieved from each of the 3 centrifugation forces, as well as the supernatant (*Cytosol*) of the 100 k ultracentrifugation, were then subject to electrophoresis analyses. The endogenous *cis*-Golgi network protein GM130 serves as the specific marker for the Golgi apparatus of HEK293T cells. (*Right*) Densitometric quantification of the ratio of the protein signal intensity at the 100 k fraction to that at the 1 k fraction (100 k/1 k) (*n* = 3). No statistical comparisons were preformed; (**B**) (*Left panels*) Representative immunoblots showing the differential centrifugation pattern of Flag-CLC-1 in the absence or presence of Myc-FKBP8 co-expression. (*Right panels*) Quantification of the relative distribution (with respect to the total signal) in the three pellet fractions for membrane-associated proteins analyzed on the gel to the left; (**C**) Statistical analyses of the 100 k/1 k ratio for the indicated membrane-associated proteins in the absence or presence of Myc-FKBP8 (*n* = 3); (**D**) Representative differential centrifugation pattern of Myc-FKBP8 in the absence or presence of Flag-CLC-1 co-expression; (**E**) Statistical analyses of the 100 k/1 k ratio for the indicated membrane-associated proteins in the absence or presence of Flag-CLC-1 (*n* = 3). The asterisk denotes a significant difference from the control (*, *t*-test: *p* < 0.05).

If FKBP8 can indeed traffic from the ER to the plasma membrane, then a portion of the co-chaperone should also be present at the Golgi complex. Although the Golgi complex is associated with a lower density relative to the ER and the plasma membrane, it is not feasible to distinguish protein distribution between the ER and the Golgi complex using standard sucrose gradient fractionation [32,33]. Instead, differential centrifugation, a fractionation analysis based on different sedimentation rates in response to increasing centrifugal force cycles (e.g., from 1000 to 100,000× *g*), has been commonly applied to differentiate the localization of ER, plasma, and Golgi membrane proteins [38,39]. For instance, compared to their Golgi counterpart, a significantly higher fraction of ER membrane proteins is found in the pellets of 1000 to 15,000× *g* centrifugation forces [38,39]. Therefore, we went on to perform differential centrifugation whereby cell homogenates were separated into 4 fractions by different centrifugal force cycles. Figure 3A exemplifies differential centrifugation analyses of lysates from HEK293T cells over-expressing GFP-tagged *trans*-Golgi network 38 (GFP-TGN38), a resident integral membrane proteins of the *trans*-Golgi network [40]. In agreement with the high-sedimentation rate of ER, calnexin was retrieved from the pellets of 1000× *g* (1 k) and 10,000× *g* (10 k), but virtually not from those of 100,000× *g* (100 k), centrifugation forces. The plasma membrane protein cadherin was present in the pellets of 1 k, 10 k, and 100 k centrifugation forces, with the strongest signal detected at the 10 k fraction. In comparison, Na^+^-K^+^-ATPase, another endogenous plasma membrane protein in HEK293T cells, displayed a more comparable distribution between 10 k and 100 k fractions, along with a minimal presence at the 1 k. Endogenous GM130, a peripheral membrane protein at the *cis*-Golgi network [41], was also found at all three pellet fractions, whereas the *trans*-Golgi-localized GFP-TGN38 was preferentially harvested from the 100 k pellet. By contrast, the cytosolic protein GAPDH remained in the supernatant of 100 k centrifugation force. To quantitatively compare the relative propensity for Golgi localization of these proteins, we then calculated the ratio of protein signal intensity at the 100 k fraction to that at the 1 k fraction [100 k/1 k]: the Golgi proteins GFP-TGN38 and GM130 were associated the highest mean 100 k/1 k ratios (~8.11 and 2.21, respectively), while Na^+^-K^+^-ATPase and cadherin showing intermediate values (~1.83 and 0.64, respectively), and calnexin the lowest (~0.01) (Figure 3A).

Like cadherin, Flag-CLC-1 was detected in the pellets of all three centrifugation forces, with the 10 k fraction showing the strongest signal (Figure 3B). In the presence of Myc-FKBP8 co-expression, there was a notable reduction of Flag-CLC-1 signal at the 1 k fraction, as well as a parallel enhancement at the 100 k fraction (Figure 3B), resulting in more than a 2-fold increase in the mean 100 k/1 k ratio, from about 0.77 to about 1.60 (Figure 3C). In comparison, no measurable effect was found on the mean 100 k/1 k ratios of cadherin, calnexin, and GM130 (Figure 3C). These observations are consistent with the notion that FKBP8 facilitates ER-exiting of the CLC-1 channel.

In direct contrast to the absence of calnexin in the 100 k pellet, Myc-FKBP8 was present in the pellets of all three centrifugation forces (Figure 3D), leading to a mean 100 k/1 k ratio from about 0.46 (Figure 3E), which is more than 20-fold higher than that of calnexin (~0.02) (Figure 3E). Importantly, co-expression with Myc-CLC-1 further increased the mean 100 k/1 k ratio of Myc-FKBP8 by almost 2-fold to about 0.88, with no discernible effect on that of cadherin, calnexin, or GM130 (Figure 3E). The foregoing data provide another line of evidence supporting the idea that a fraction of FKBP8 may be president at the Golgi complex, and that over-expressing CLC-1 promotes ER-to-Golgi transition of FKBP8. 

**Figure 4:**
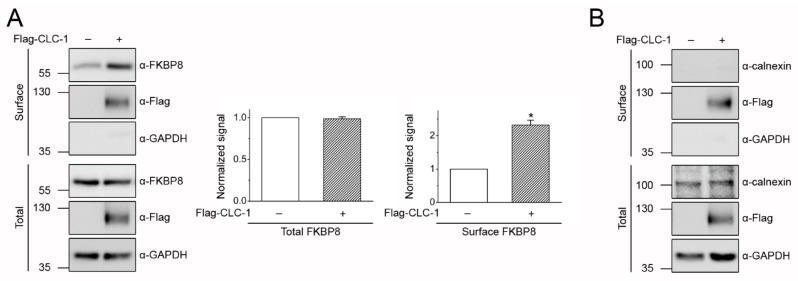

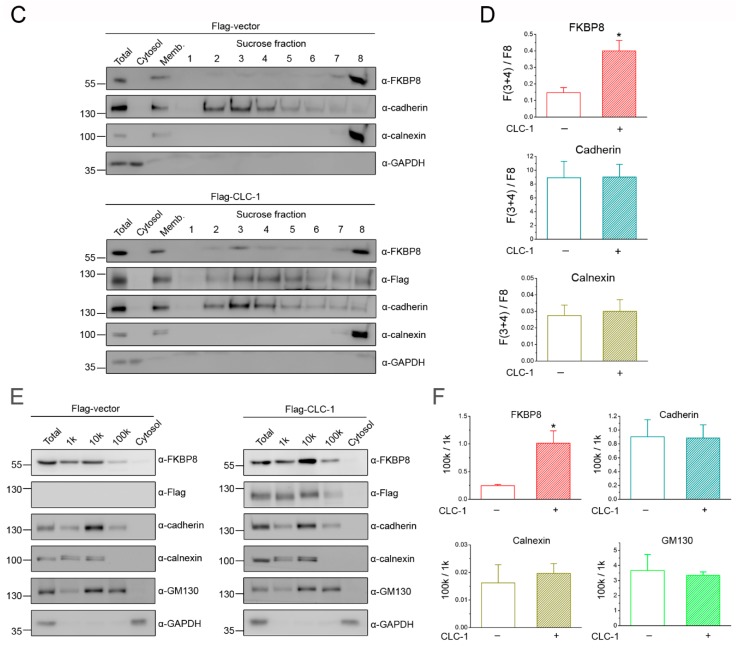
Subcellular localization of endogenous FKBP8 in HEK293T cells. (**A**) Surface biotinylation analysis of endogenous FKBP8 in the absence or presence of Flag-CLC-1 over-expression (*n* = 3); (**B**) Representative biotinylation result of endogenous calnexin in the absence or presence of Flag-CLC-1 over-expression; (**C**) Representative sucrose density gradient centrifugation pattern of endogenous FKBP8 in HEK293T cells in the absence (*Flag-vector*) or presence (*Flag-CLC-1*) of Flag-CLC-1 over-expression; (**D**) Statistical analyses of the F(3+4)/F8 ratio for the indicated membrane-associated proteins in the absence or presence of Flag-CLC-1 (*n* = 4); (**E**) Representative differential centrifugation pattern of endogenous FKBP8 in HEK293T cells in the absence or presence of Flag-CLC-1 over-expression; (**F**) Statistical analyses of the 100 k/1 k ratio for the indicated membrane-associated proteins in the absence or presence of Flag-CLC-1 (*n* = 4). The asterisk denotes a significant difference from the control (*, *t*-test: *p* < 0.05).

All the preceding localization experiments involve over-expressed Myc-FKBP8 instead of endogenous FKBP8, raising the concern that our conclusions may be based on artifacts instigated by protein over-expression. To address this critical issue, we first examined the subcellular localization of endogenous FKBP8 in HEK293T cells. Figure 4A illustrates that a significant fraction of endogenous FKBP8 in HEK293T cells was indeed detected by surface biotinylation, and that over-expression of Flag-CLC-1 led to about a 2-fold increase in the surface localization of endogenous FKBP8. By contrast, regardless of whether Flag-CLC-1 was over-expressed in HEK293T cells, the endogenous ER-membrane protein calnexin was not discernibly detected by surface biotinylation (Figure 4B). Furthermore, sucrose density gradient analyses in Figure 4C demonstrate that a faint signal of endogenous FKBP8 was present at fraction 3, with a mean F(3+4)/F8 ratio of about 0.15 (Figure 4D), which is significantly higher than that of calnexin (~0.02) (Figure 4D), but lower than that of over-expressed Myc-FKBP8 (~0.34) (Figure 2D). Importantly, Flag-CLC-1 co-expression induced a substantial increase of endogenous FKBP8 signal at fraction 3 (Figure 4C), resulting in more than 2-fold increase in the mean F(3+4)/F8 ratio to about 0.4 (Figure 4D). In addition, differential centrifugation analyses detected considerable FKBP8 signal in the 100 k pellet (Figure 4E). The estimated mean 100 k/1 k ratio of endogenous FKBP8 was about 0.25 (Figure 4F), which is lower than that of Myc-FKBP8 (~0.46) (Figure 3E), but distinctly higher than that of calnexin (~0.02) (Figure 4F). Notably, Flag-CLC-1 co-expression also promoted 100 k-localization of endogenous FKBP8 (Figure 4E), leading to more than a 4-fold increase of the mean 100 k/1 k ratio to about 1.01 (Figure 4F), which is comparable to that of Myc-FKBP8 (~0.88) under the same co-expression condition (Figure 3E). Taken together, we infer that, in addition to its known ER localization, endogenous FKBP8 in HEK293T cells is also resident at both the plasma membrane and the Golgi complex. Significantly, like Myc-FKBP8, both plasma membrane- and Golgi complex-localizations of endogenous FKBP8 are remarkably enhanced in the presence of the substrate protein CLC-1.

**Figure 5:**
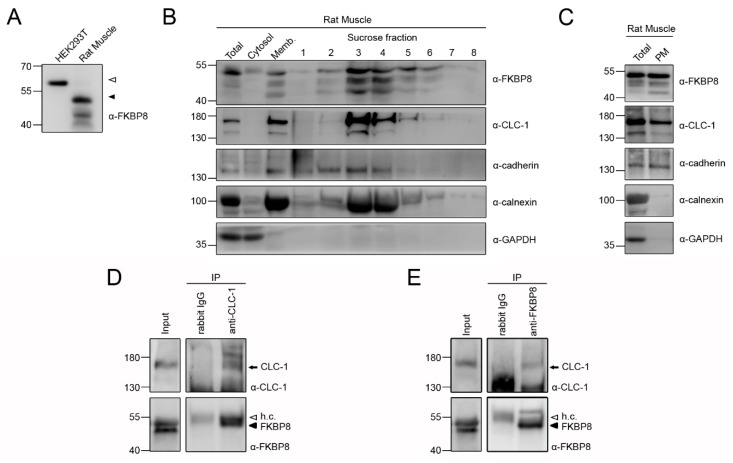
Plasma membrane-localization of endogenous FKBP8 in skeletal muscle. (**A**) Representative protein signals of endogenous FKBP8 in human (HEK293T) cells (*open triangle*) and rat skeletal muscle (*filled triangle*); (**B**) Representative sucrose fractionation pattern of endogenous FKBP8, CLC-1, cadherin, calnexin, and GAPDH in rat skeletal muscle; (**C**) Representative immunoblot showing the presence of rat muscle CLC-1, cadherin, and FBKP8 at the plasma membrane. Plasma membrane fraction (*PM*) was isolated with an aqueous two-phase system. Note that GAPDH and calnexin are found in the total protein fraction (*Total*), but not in the plasma membrane fraction; (**D**,**E**) Co-immunoprecipitation of FKBP8 and CLC-1 in skeletal muscle. Muscle lysates were subject to immunoprecipitation (*IP*) with the anti-CLC-1 (**D**) or anti-FKBP8 (**E**) antibody, followed by immunoblotting with the indicated antibodies. Rabbit IgG was employed to verify the specificity of immunoprecipitation. Corresponding expression levels of CLC-1 and FKBP8 in the lysates are shown in the *Input* lane. Input represents about 10% of the total protein used for immunoprecipitation. *h.c.*: IgG heavy chain.

In light of the fact that CLC-1 is a skeletal muscle-specific ion channel, we went on to investigate whether endogenous FKBP8 in rat skeletal muscle may also be present at the plasma membrane. Consistent with the presence of a 10-amino acid difference in protein size between the two FKBP8 homologues, the apparent molecular weight of rat FKBP8 was relatively lower than that of its human counterpart (Figure 5A). Figure 5B depicts a representative sucrose density gradient analysis of endogenous proteins in rat skeletal muscle. As expected, endogenous CLC-1 and cadherin were detected at fractions 3–5 and 2–4, respectively. However, in direct contrast to its virtually exclusive localization at fraction 8 in HEK293T cells, endogenous calnexin in skeletal muscle was present at the fractions 2–6, with a minimal signal at fraction 8. A similar widespread distribution pattern in skeletal muscle was also found for endogenous FKBP8. The presence of this overlap between ER and plasma membrane proteins in sucrose density gradient may be ascribed to the structural complexity of ER (sarcoplasmic reticulum, SR) in skeletal muscle. The SR in skeletal muscle comprises two domains interconnected in a network-like manner: the “light” longitudinal SR and the “heavy” junctional SR, with the latter being associated with the plasma membrane (sarcolemma and transverse tubule) [42]. Moreover, calnexin and BiP, two of the most common ER-resident proteins, were found to be widely distributed within both longitudinal and junctional SR [43]. In agreement with these notions, sucrose density gradient *per se* does not appear to be effective in separating SR and plasma membranes in skeletal muscle; rather, additional affinity purification procedures are required to isolate the plasma membrane fraction [44]. We therefore chose an aqueous two-phase partition system to isolate the plasma membrane of rat skeletal muscle [45,46]. Figure 5C demonstrates that both endogenous CLC-1 and cadherin were readily detected at the surface membrane fraction. Most importantly, endogenous FKBP8, but not calnexin, was present at the plasma membrane of skeletal muscle. In addition, co-immunoprecipitation analyses confirmed the interaction of endogenous FKBP8 and CLC-1 in skeletal muscle (Figure 5D,E). 

### 2.3. Regulation of Surface CLC-1 Stability by FKBP8

**Figure 6:**
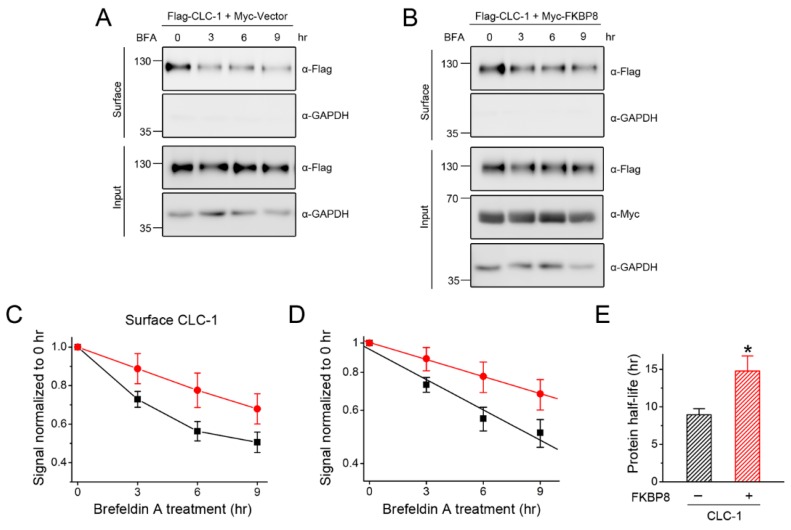
FKBP8 promotes surface protein stability. (**A**,**B**) Representative immunoblots showing the time course of surface Flag-CLC-1 protein turn-over in the absence (**A**) or presence (**B**) of Myc-FKBP8 over-expression. Transfected HEK293T cells were subject to the indicated durations of treatment with the Golgi trafficking inhibitor brefeldin A (BFA) (5 μg/mL), followed by surface biotinylation analyses; (**C**–**E**) Quantification of surface Flag-CLC-1 protein stability in the absence (*black*) or presence (*red*) of Myc-FKBP8 over-expression (*n* = 6–8); (**C**) Linear plot of the turn-over kinetics of surface CLC-1 protein densities. Protein density was standardized as the ratio of surface Flag-CLC-1 signal to the cognate total GAPDH signal, followed by normalization to the corresponding control at 0 hr; (**D**) Semi-logarithmic plot of the same data points, followed by linear-regression analyses (*solid lines*); (**E**) Statistical analyses of surface CLC-1 protein half-life. The surface protein turn-over time course determined from each experiment was individually plotted on a semi-logarithmic scale for linear-regression analysis, and independent protein half-life values were pooled together for statistical comparisons. The asterisk denotes a significant difference from the control (*, *t*-test: *p* < 0.05).

Having established the co-localization of FKBP8 and CLC-1 at the plasma membrane, we then assessed the significance of this co-localization on protein stability of surface CLC-1. By applying the protein translation inhibitor cycloheximide, we have previously shown that FKBP8 over-expression prolonged the half-life of total CLC-1 protein by more than 50% [19]. One plausible approach to evaluating protein half-life of surface CLC-1 would involve performing cycloheximide chase analyses of surface biotinylated CLC-1 signal. Nonetheless, given that FKBP8 is known to promote membrane trafficking of CLC-1 [19], the cycloheximide chase experiment does not appear to be appropriate for assessing the effect of the co-chaperone on surface CLC-1 stability. Instead, we employed brefeldin A (BFA), which inhibits forward trafficking of proteins from the ER to the Golgi [47], to investigate the effect of FKBP8 co-expression on protein stability of plasma membrane-resident CLC-1. In other words, as a result of BFA-induced blockade of forward trafficking, the time course of the reduction in surface biotinylated CLC-1 protein in response to increasing BFA treatment durations reflects the turnover rate of CLC-1 channels at the plasma membrane, a quantitative analysis known as BFA chase assay [48]. Figure 6 exemplifies the BFA chase response of surface CLC-1 protein: in the presence of FKBP8, the estimated protein half-life of surface CLC-1 dramatically increased from about 8.4 to about 14.8 h, implying that FKBP8 may effectively enhance protein stability of CLC-1 at the plasma membrane. Consistent with this notion, our previous functional analyses using patch clamp recording demonstrated that co-expression with FKBP8 substantially enhanced CLC-1 current level [19].

## 3. Discussion

FKBP8, a member of the FK506-binding protein family, is a membrane-anchored immunophilin that serves as a potential peptidyl-prolyl *cis-trans* isomerase in the ER quality control system [21,22,24,27,49,50]. Traditionally, FKBP8 is regarded as an internal membrane protein resident at the ER and the mitochondria [26,35,36,37]. In the current study, we employed various biochemical techniques to provide the novel evidence indicating that FKBP8 may also be localized at the Golgi complex and the plasma membrane. Nevertheless, based on the mean F(3+4)/F8 ratios derived from our sucrose density gradient analyses, FKBP8 (~0.12–0.34) does display a markedly reduced propensity for cell surface localization, as compared to typical plasma membrane proteins such as cadherin (~6.31–9.02), CLC-1 (~1.87–2.92), and K_V_1.1 (~0.66–1.23). 

We previously demonstrated that the molecular chaperones Hsc70 and Hsp90β, as well as the co-chaperones HOP, Aha1, and FKBP8, are binding partners of human CLC-1 channel [19]. In the ER quality control system of CLC-1 protein, Hsc70 and HOP appear to mediate a facilitating process at an early stage, followed by a Hsp90β cycle that entails a concerted work by Aha1, Hsp90β, and FKBP8 to further promote CLC-1 folding [19]. Hsp90β and FKBP8 may additionally contribute to ER-associated degradation of misfolded CLC-1 by interacting with the Cullin 4A/B E3 ubiquitin ligase. Importantly, compared to the other molecular chaperones and co-chaperones, FKBP8 displays a profoundly significant effect in increasing membrane expression of CLC-1 protein. This prominent increase in surface expression clearly highlights the notion that FKBP8 is distinctively effective in assisting ER exiting of CLC-1. In the present study, we further demonstrated that FKBP8 may be co-localized with CLC-1 at both the Golgi complex and the plasma membrane. Interestingly, co-expression with the substrate protein CLC-1 results in enhanced Golgi- and plasma membrane-localization of FKBP8. Taken together, we propose that, in addition to serving as an ER-resident co-chaperone, FKBP8 may usher the membrane trafficking process of CLC-1 from the ER, through the Golgi complex, and eventually to the plasma membrane. Remarkably, our data also indicate that FKBP8 effectively prolongs CLC-1 protein half-life at the cell surface. Consequently, the FKBP8-induced increase in surface expression may in part be accounted for by enhanced CLC-1 protein stability at the plasma membrane. 

Emerging evidence supports the notion that membrane proteins are subject to peripheral quality control whereby protein homeostasis at the cell surface is stringently controlled by protein conformation surveillance and endosomal-lysosomal degradation systems [51,52,53]. Disruption in protein stability at the plasma membrane has been implicated in many human diseases. For example, CFTR ∆F508, the most common cystic fibrosis-causing mutation [54,55], is associated with enhanced lysosomal degradation [56,57]. A similar defect in surface stability has also been demonstrated for the myotonia congenita-causing CLC-1 A531V mutation [13]. Despite its well documented function in ER quality control [20,21,22], FKBP8 does not seem to contribute to the peripheral quality control of CFTR [56]. In this report, we observed enhanced surface CLC-1 stability in the presence of FKBP8 over-expression. Hence, to the best of our knowledge, we provide the first direct evidence suggesting that FKBP8 may play an essential role in the peripheral quality control of membrane protein.

Although we failed to detect CLC-1-induced surface biotinylation signals of Aha1, HOP, Hsp90β, and Hsc70, we cannot discount the possibility that some of these molecular chaperones and co-chaperones may still contribute to CLC-1 peripheral quality control, as has been suggested for CFTR [56]. Further investigation is required to understand how FKBP8 interacts with chaperones/co-chaperones, as well as counteracting E3 ubiquitin ligases, to maintain CLC-1 protein homeostasis at the plasma membrane. It is also important in the future to ascertain how FKBP8 assists CLC-1 trafficking in post-ER compartments. In addition, ER-related chaperones and co-chaperones are required for the assembly of ion channel subunits [58,59]. Hence it will be interesting to address whether FKBP8 may facilitate the dimerization of CLC-1 subunits at the ER. 

Defective protein stability in CLC-1 channel has been associated with the loss-of-function neuromuscular diseases myotonia congenita [12,13]. Emerging therapeutic strategies for human diseases focus on the identification of novel chemical interventions effective in adjusting anomalous protein biosynthesis networks to restore normal physiology [60,61]. For myotonia congenita, therefore, elucidation of the molecular mechanisms governing ER and peripheral quality control systems is imperative for developing new approaches for correcting misfolded channel proteins. Our demonstration of the important role of FKBP8 in peripheral quality control may shed light on the search for pharmacological agents potentially effective in overcoming excessive clearance of the CLC-1 channel at the plasma membrane.

## 4. Materials and Methods

### 4.1. cDNA Constructs

Flag-CLC-1 was generated by subcloning human CLC-1 into the pFlag-CMV2 vector (Sigma-Aldrich, St. Louis, MO, USA) [18]. Myc-FKBP8 was created by swapping mouse FKBP8 into the pcDNA3.1-Myc vector (Sigma-Aldrich) [19]. Additional cDNA constructs employed in this study are pcDNA3.1-Myc mouse Aha1, pcDNA3-HA rat HOP, pcDNA5-V5 human Hsc70 (Addgene 19514, Watertown, MA, USA), pcDNA3-HA human Hsp90β (Addgene 22847), and pEGFP-rat TGN38 (kindly provided by Dr. Fan-Jen Lee, National Taiwan University, Taipei, Taiwan).

### 4.2. Cell Culture and DNA Transfection

Human embryonic kidney (HEK) 293T cells were grown in Dulbecco’s modified Eagle’s medium (DMEM) (Invitrogen, Carlsbad, CA, USA) supplemented with 10% heat-inactivated fetal bovine serum (Hyclone, Logan, UT, USA), 2 mM glutamine, 100 units/mL penicillin, and 50 μg/mL streptomycin, and were maintained at 37 °C in a humidified incubator with 95% air and 5% CO_2_. Transient transfection was performed with the calcium phosphate method [62]. Cells were plated onto 12-well plates or 10-cm dishes (for biochemical experiments), or poly-d-lysine-coated coverslips in 24-well plates (for immunofluorescence experiments) 24 hrs before transfection. The amount of cDNA used in each well was about 200 (for expression) to 500 (for immunoprecipitation) ng/mL. The molar ratio for co-transfection was 1:1. After 6-hr incubation at 37 °C, the medium was changed and the culture cells were maintained in the 37 °C incubator for 24–48 hrs before being used for biochemical or immunofluorescence experiments. Where indicated, brefeldin A (Sigma-Aldrich) was added to the culture medium. 

### 4.3. Immunoblotting

Cells were washed twice with ice-cold phosphate buffered saline (PBS) [(in mM) 137 NaCl, 2.7 KCl, 4.3 Na_2_HPO_4_·2H_2_O, 1.4 KH_2_PO_4_, pH 7.3] supplemented with 2 mM EDTA, and re-suspended in a lysis buffer [(in mM) 150 NaCl, 5 EDTA, 50 Tris-HCl pH7.6, 1% Triton X-100) containing protease inhibitor cocktail (Roche Applied Science, Mannhaim, Germany). The Laemmli sample buffer was added to the lysates, which in turn were sonicated on ice (3 times for 5 sec each) and heated at 70 °C for 5 min. Samples were then separated by 7.5–10% SDS-PAGE, electrophoretically transferred to nitrocellulose membranes, and detected using rabbit anti-Aha1 (1:2500; Thermo Scientific, Waltham, MA, USA), mouse anti-cadherin (1:1000; Abcam, Cambridge, MA, USA), mouse anti-calnexin (1:1000; Santa Cruz Biotechnology, Dallas, TX, USA), rabbit anti-CLC-1 (1:1000; Alomone, Jerusalem, Israel), rabbit anti-Flag (1:5000; Sigma-Aldrich), rabbit anti-FKBP8 (1:5000; GeneTex, Hsinchu, Taiwan), rabbit anti-GAPDH (1:8000; GeneTex), rat anti-HA (1:5000; Roche Applied Science), rabbit anti-GFP (1:5000; Abcam), mouse anti-GM130 (1:1000; BD Biosciences, San Jose, CA, USA), rabbit anti-HOP (1:10,000; Abcam), rabbit anti-Hsc70 (1:750; Abcam), rabbit anti- Hsp90β (1:500; Abcam), mouse anti-Myc (clone 9E10), or mouse anti-Na^+^-K^+^-ATPase (1:1000; Thermo Scientific) antibodies. Blots were exposed to horseradish peroxidase-conjugated anti-mouse/rabbit IgG (1:5000; Jackson ImmunoResearch, West Grove, PA, USA) or goat anti-rat IgG (1:5000; Santa Cruz Biotechnology), and then revealed by an enhanced chemiluminescence detection system (Thermo Scientific). Results shown are representative of at least 3 independent experiments. Densitometric scans of immunoblots were quantified by using ImageJ (National Institute of Health, Bethesda, MD, USA).

### 4.4. Cell Surface Biotinylation 

Cells were rinsed with ice-cold Dulbecco’s phosphate buffered saline (D-PBS) (Sigma-Aldrich) supplemented with 0.5 mM CaCl_2_, 2 mM MgCl_2_, incubated in 1 mg/mL sulfo-NHS-LC-biotin (Thermo Scientific) in D-PBS at 4 °C for 1 hr with gentle rocking on an orbital shaker, and subject to a quenching procedure by removing the biotin reagents and rinsing 3 times with 100 mM glycine in PBS, followed by once in Tris buffered saline (TBS) [(in mM) 20 Tris-HCl, 150 NaCl, pH 7.4]. Cells were solubilized in ice-cold lysis buffer [(in mM) 150 NaCl, 50 Tris-HCl, 1% Triton X-100, 5 EDTA, 1 phenylmethylsulfonyl fluoride (PMSF), pH 7.6] supplemented with the protease inhibitor cocktail. Insolubilized materials were removed by centrifugation at 4 °C. Solubilized cell lysates were incubated overnight at 4 °C with streptavidin-agarose beads (Thermo Scientific). Beads were washed once in the lysis buffer, followed by twice in a high-salt buffer [(in mM) 500 NaCl, 5 EDTA, 50 Tris-HCl, pH7.6, 0.1% Triton X-100] and once in a low-salt buffer [(in mM) 2 EDTA, 10 Tris-HCl, pH7.6, 0.1% Triton X-100]. Biotin-streptavidin complexes were eluted from the beads by heating at 70 °C for 5 min in the Laemmli sample buffer. 

For quantitative analyses, cell lysates from biotinylated intact cells were subject to either direct immunoblotting analyses (*Total*) or streptavidin pull-down prior to immunoblotting (*Surface*). Specifically, during sample loading for SDS-PAGE, the amount of lysates loaded in the *Total* lane represents about 8% of that used for streptavidin pull-down and thereafter loading in the *Surface* lane. For both *Total* and *Surface* signals, the protein density was standardized as the ratio to the cognate total GAPDH signal. The membrane trafficking efficiency (*Surface/total*) was determined as the ratio of surface protein density to the corresponding total protein density. In all cases, the mean value was normalized to that of the corresponding vector control.

### 4.5. Subcellular Fractionation Analyses

Subcellular fractionation protocols were modified from our previously published procedures [48]. In general, cells were washed with ice-cold PBS, detached from dishes with pre-cooled PBS containing 2 mM EDTA, and pelleted for 5 min at 1000× *g*. Cell pellets were suspended in hypotonic buffer (as specified below) and homogenized by 20 strokes in a Dounce homogenizer, followed by specific processes for either sucrose fractionation or differential centrifugation.

For sucrose gradient fractionation, the hypotonic buffer comprises (in mM) 2 EDTA, 2 EGTA, 6 MgCl_2_, 20 HEPES, pH 7.4, and 1 PMSF, supplemented with protease inhibitor cocktail. Cell lysates were further homogenized by stroking in a 23 G needle for 10 times, and then centrifuged at 1000× *g* at 4 °C for 5 min to remove undisrupted cells. A portion of the supernatant was collected as the “total homogenate”, and the rest was subject to 100,000× *g* centrifugation for 90 min, thereby separating into the “cytosol” (supernatant) and the “membrane” (pellet) fractions. Pellets were rinsed with and sonicated in hypotonic buffer, followed by suspension in hypotonic buffer supplemented with 2 M sucrose to achieve a 0.2 M final sucrose concentration. Membrane lysates were then loaded on the top of a discontinuous sucrose gradient [2 M (0.4 mL), 1.5 M (0.75 mL), 1.35 M (0.75 mL), 1.2 M (0.75 mL), 0.9 M (0.5 mL), and 0.5 M (0.5 mL) in hypotonic buffer], and subject to a 32,000× *g* centrifugation for 16 hr. Fractions were collected from the top of the gradient, including one 1-mL and seven 0.5-mL fractions.

For differential centrifugation, the hypotonic buffer comprises (in mM) 20 Tris-HCl, 2 DTT, 10 EDTA, pH 7.9, and 1 PMSF supplemented with protease inhibitor cocktail. Cell lysates were further homogenized by stroking in a 26 G needle for 10 times, re-suspended in 0.2 M sucrose in hypotonic solution, and then centrifuged at 100× *g* at 4 °C for 10 min to remove undisrupted cells. A fraction of the supernatant was collected as the “total homogenate”, and the rest was subject to 1000× *g* centrifugation for 10 min, generating the “1 k” pellet. The supernatant was then centrifuged at 10,000× *g* for 30 min, generating the “10 k” pellet. The supernatant was further centrifuged at 100,000× *g* for 90 min, generating the “100 k” pellet. The 1/10/100 k pellet was rinsed with hypotonic buffer, followed by suspension and sonication in ice-cold lysis buffer [(in mM) 100 NaCl, 4 KCl, 2.5 EDTA, 20 NaHCO_3_, 20 Tris-HCl, pH 7.5, 1 PMSF] with 1% Triton X-100.

### 4.6. Isolation of Plasma Membrane Proteins from Rat Skeletal Muscle 

Animals were anesthetized and sacrificed in accordance with the Guidelines for the Care and Use of Mammals in Neuroscience and Behavioral Research (National Research Council 2003), as well as following procedures approved by the Institutional Animal Care and Use Committee (IACUC) of College of Medicine, National Taiwan University. Vastus lateralis muscle fibers were dissected from adult Wistar rats as described previously [18]. Aqueous two-phase purification of plasma membrane was accomplished by using the Plasma Membrane Protein Extraction Kit (Abcam). Briefly, muscle tissues were homogenized using the hypotonic buffer for sucrose fractionation (0.05 g muscle in 2 mL buffer). Muscle lysates were further treated with the Homogenize Buffer Mix, followed by micro-centrifugation at 1000× *g* and then 100,000× *g* at 4 °C for 5 and 90 min, respectively. The pellet, which contains total cellular membrane proteins, was re-suspended in the Upper Phase Solution, incubated on ice for 5 min, mixed with the Lower Phase Solution, and micro-centrifuged at 1000× *g* for 5 min. The upper phase of the solution mix was carefully collected, micro-centrifuged at 1000× *g* for 5 min, diluted in water, kept on ice for 5 min, and micro-centrifuged at top speed for 10 min. The pellet, which contains plasma membrane proteins, was dissolved in 0.5% Triton X-100 in PBS. 

### 4.7. Statistical Analyses 

All values were presented as mean ± SEM. The significance of the difference between two means was tested using Student’s *t* test, whereas means from multiple groups were compared using one-way ANOVA. All statistical analyses were performed with Origin 7.0 (OriginLab, Northampton, MA, USA).

## Abbreviations

Aha1	activator of Hsp90 ATPase homolog 1
BFA	brefeldin A
CFTR	cystic fibrosis transmembrane conductance regulator
Cl-	chloride
DMEM	Dulbecco’s modified Eagle’s medium
D-PBS	Dulbecco’s phosphate buffered saline
DTT	dithiothreitol
ER	endoplasmic reticulum
FKBP8	FK506-binding protein 8
GAPDH	glyceraldehyde 3-phosphate dehydrogenase
GST	glutathione S-transferase
HEK	human embryonic kidney
HOP	Hsp70/Hsp90 organizing protein
Hsc70	heat shock cognate protein 70
Hsc70	heat shock cognate protein 70
Hsp90β	heat shock protein 90β
PBS	phosphate buffered saline
PMSF	phenylmethylsulfonyl fluoride
SR	sarcoplasmic reticulum
TBS	Tris buffered saline
TGN38	trans-Golgi network 38
